# Impacts of sea level rise and climate change on coastal plant species in the central California coast

**DOI:** 10.7717/peerj.958

**Published:** 2015-05-12

**Authors:** Kendra L. Garner, Michelle Y. Chang, Matthew T. Fulda, Jonathan A. Berlin, Rachel E. Freed, Melissa M. Soo-Hoo, Dave L. Revell, Makihiko Ikegami, Lorraine E. Flint, Alan L. Flint, Bruce E. Kendall

**Affiliations:** 1 Bren School of Environmental Science & Management, University of California, Santa Barbara, USA; 2 Revell Coastal, LLC, Santa Cruz, CA, USA; 3 U.S. Geological Survey California Water Science Center, Sacramento, CA, USA

**Keywords:** Sea level rise, Plants, Coastal, Inundation, Flooding, Erosion, Habitat

## Abstract

Local increases in sea level caused by global climate change pose a significant threat to the persistence of many coastal plant species through exacerbating inundation, flooding, and erosion. In addition to sea level rise (SLR), climate changes in the form of air temperature and precipitation regimes will also alter habitats of coastal plant species. Although numerous studies have analyzed the effect of climate change on future habitats through species distribution models (SDMs), none have incorporated the threat of exposure to SLR. We developed a model that quantified the effect of both SLR and climate change on habitat for 88 rare coastal plant species in San Luis Obispo, Santa Barbara, and Ventura Counties, California, USA (an area of 23,948 km^2^). Our SLR model projects that by the year 2100, 60 of the 88 species will be threatened by SLR. We found that the probability of being threatened by SLR strongly correlates with a species’ area, elevation, and distance from the coast, and that 10 species could lose their entire current habitat in the study region. We modeled the habitat suitability of these 10 species under future climate using a species distribution model (SDM). Our SDM projects that 4 of the 10 species will lose all suitable current habitats in the region as a result of climate change. While SLR accounts for up to 9.2 km^2^ loss in habitat, climate change accounts for habitat suitability changes ranging from a loss of 1,439 km^2^ for one species to a gain of 9,795 km^2^ for another species. For three species, SLR is projected to reduce future suitable area by as much as 28% of total area. This suggests that while SLR poses a higher risk, climate changes in precipitation and air temperature represents a lesser known but potentially larger risk and a small cumulative effect from both.

## Introduction

The average global sea level is rising, with evidence to suggest that the rate is accelerating (IPCC, 2007; Titus et al., 2009; Nicholls & Cazenave, 2010). As increasing atmospheric concentrations of greenhouse gases warm the atmosphere and oceans, sea level is rising due to thermal expansion of waters and the melting of glaciers and ice sheets (Nicholls & Cazenave, 2010). While global mean sea level has been gradually increasing for at least 20,000 years, this trend has accelerated in the last 15 to 20 years in response to climate change (IPCC, 2007). According to recent projections, global mean sea level could rise as much as 32 cm in the next 40 years and rise 75 to 190 cm over the next century (Pfeffer, Harper & O’Neel, 2008; Vermeer & Rahmstorf, 2009; Nicholls & Cazenave, 2010; Revell et al., 2011; Slangen et al., 2012). Rising sea level and the potential for stronger storms pose an increasing threat to coastal communities, infrastructure, beaches, and ecosystems.

Given the dynamic nature of the coastal zone, the response of coastal areas to sea level rise (SLR) is more complex than simple inundation. In addition to inundating low-lying areas, rising sea levels can increase flooding events, coastal erosion, wetland loss, and saltwater intrusion into estuaries and freshwater aquifers. Certain marsh and wetland species, such as mangroves and seagrass, are projected to decline in abundance under SLR (Traill et al., 2011; Comeaux, Allison & Bianchi, 2012; Saunders et al., 2013). Moreover, climate change will likely result in altered patterns of precipitation and warmer temperatures in some coastal areas along with increasing risks of extreme high sea level events. This is expected to be especially common during high tides, particularly when exacerbated by winter storms and El Niño events (Cayan et al., 2008a). The combined effects of SLR and other climate change factors, including changes in fog, may cause rapid and irreversible coastal changes that will have significant effects on coastal habitats and species.

In the United States, climate-related changes are already being observed in the form of rising temperature and sea level, storms, early snowmelt, lengthening of growing seasons, and alterations in river flows, among others (Karl et al., 2009). Furthermore, these changes are projected to intensify over the coming century (Karl et al., 2009). Climate change in the form of increasing air temperature and varying precipitation will also affect coastal plant species in California (Hayhoe et al., 2004). Climatic factors are known to be important drivers of species’ distributions (Woodward & Williams, 1987); climate change could alter the current distribution of a species by shrinking or enlarging and ranges shifting its climatic envelope (Jones et al., 2013; Smale & Wernberg, 2013). Many coastal species are also adapted to specific temperature ranges, and an increase in temperatures will likely change the distribution of these species (Titus et al., 2009). Rare and threatened native plants are more susceptible to extinction caused by climate change due principally to their small population sizes and specific habitat requirements. Gradual migration to new habitats can be especially difficult for rare plant species with small populations, since they may be constrained by low dispersal ability, genetic diversity, and limited habitat (Maschinski et al., 2011). Furthermore, unlike more mobile species, plant migration depends on a variety of dispersal agents (Howe & Smallwood, 1982) that also may also be negatively affected by climate change. Some studies estimate that endemic plant species’ ranges may shift up to 90 miles under drastic climate change; however, the rate of movement over that distance would be far slower than the rate of climate change (Loarie et al., 2008).

Numerous studies have analyzed the effect of climate change on future habitats through species distribution modeling (SDM) (Guisan & Zimmermann, 2000; Bakkenes et al., 2002; Thomas et al., 2004; Guisan & Thuiller, 2005; Thuiller, Lavorel & Araújo, 2005), which statistically relates multiple abiotic habitat characteristics with observed occurrences of a species (Kearney & Porter, 2004; Guisan & Thuiller, 2005; Araújo & Guisan, 2006). In California, Loarie et al. (2008) estimated that approximately 66% of California’s endemic plant species may experience decreases of up to 80% in the size of their ranges within the next 100 years as a result of climate change. Although numerous studies have been published evaluating climate change effects on species distributions, few studies have incorporated the threat of exposure to SLR with species distribution under climate change (Saunders et al., 2013) and none to our knowledge have addressed the effects on a wide range of species. There is a pressing need to identify the existence of interacting effects between climate change and habitat loss and, if so, to quantify the magnitude of their impact (Mantyka-Pringle, Martin & Rhodes, 2012).

**Figure 1:**
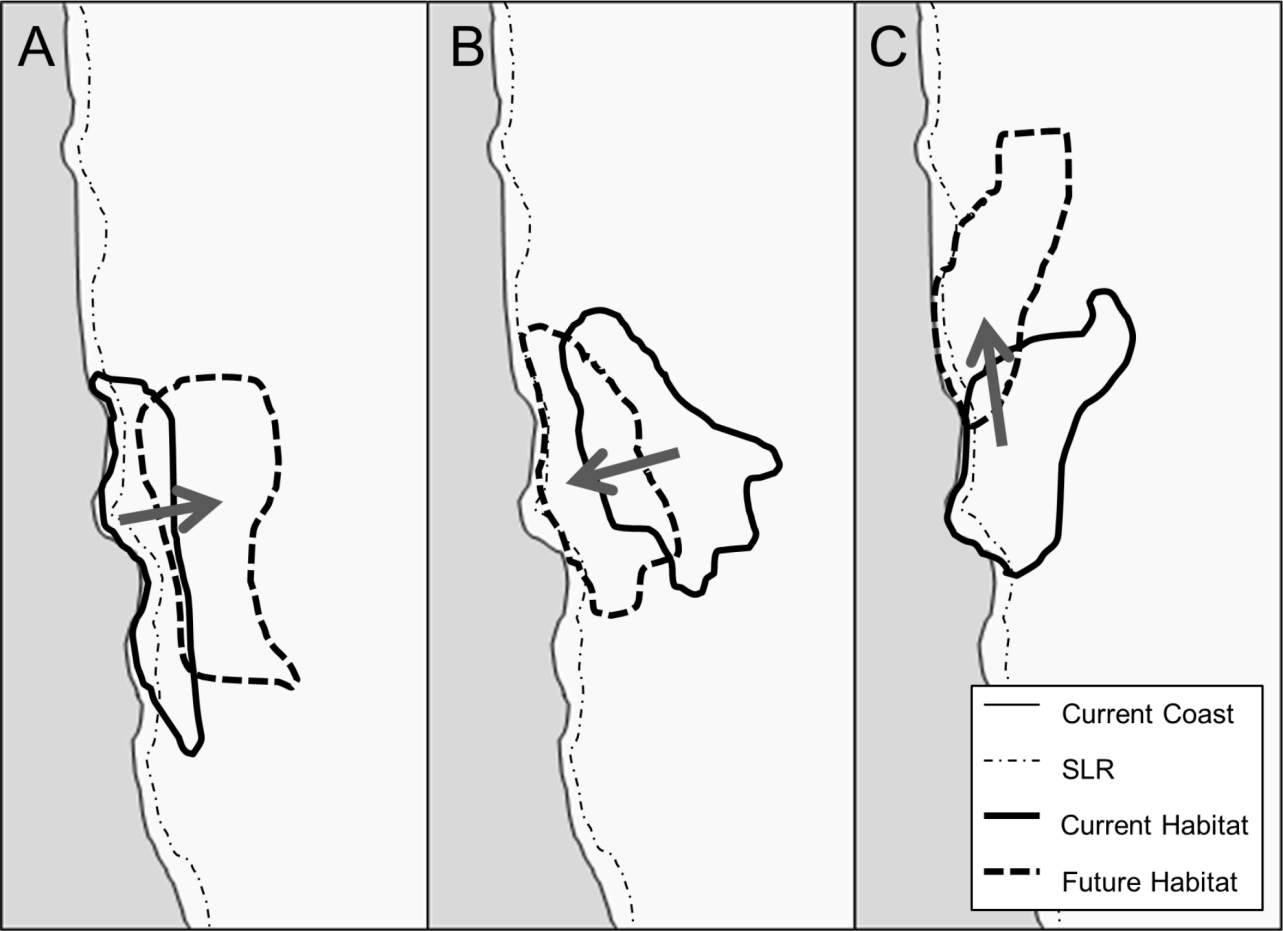
Conceptual model of suitable habitat shifts as a result of climate change and the resulting impact of SLR on that habitat. In A, climate change shifts species range away from the coast, thus decreasing the threat of SLR. In B, climate change shifts species range towards the coast, thus increasing the threat of SLR. In C, climate change shifts species range up the coast (North), thus having no significant change to the threat of SLR.

Conceptually, the combined influence of climate change and SLR may result in three distinct patterns (Fig. 1). In the first case, climate change could shift species inland and thus away from the threat of SLR (Fig. 1A). Second, climate change could shift species toward the coast, thus threatening species that would not have otherwise been affected by SLR (Fig. 1B). In the third case, climate change could shift species habitats along the coast (Loarie et al., 2008), which depending on the coastline could result in no net change in the threat of SLR to the species (Fig. 1C).

We addressed the following questions in our study: (1) What is the extent of the impact of SLR on rare plant species along the central California, USA coast; (2) Which plant characteristics are the best predictors of exposure to SLR; (3) To what extent will climate change shift the current habitat of rare coastal plant species in the future; (4) What is the relative impact of climate change compared to SLR on the habitat of species?

**Figure 2:**
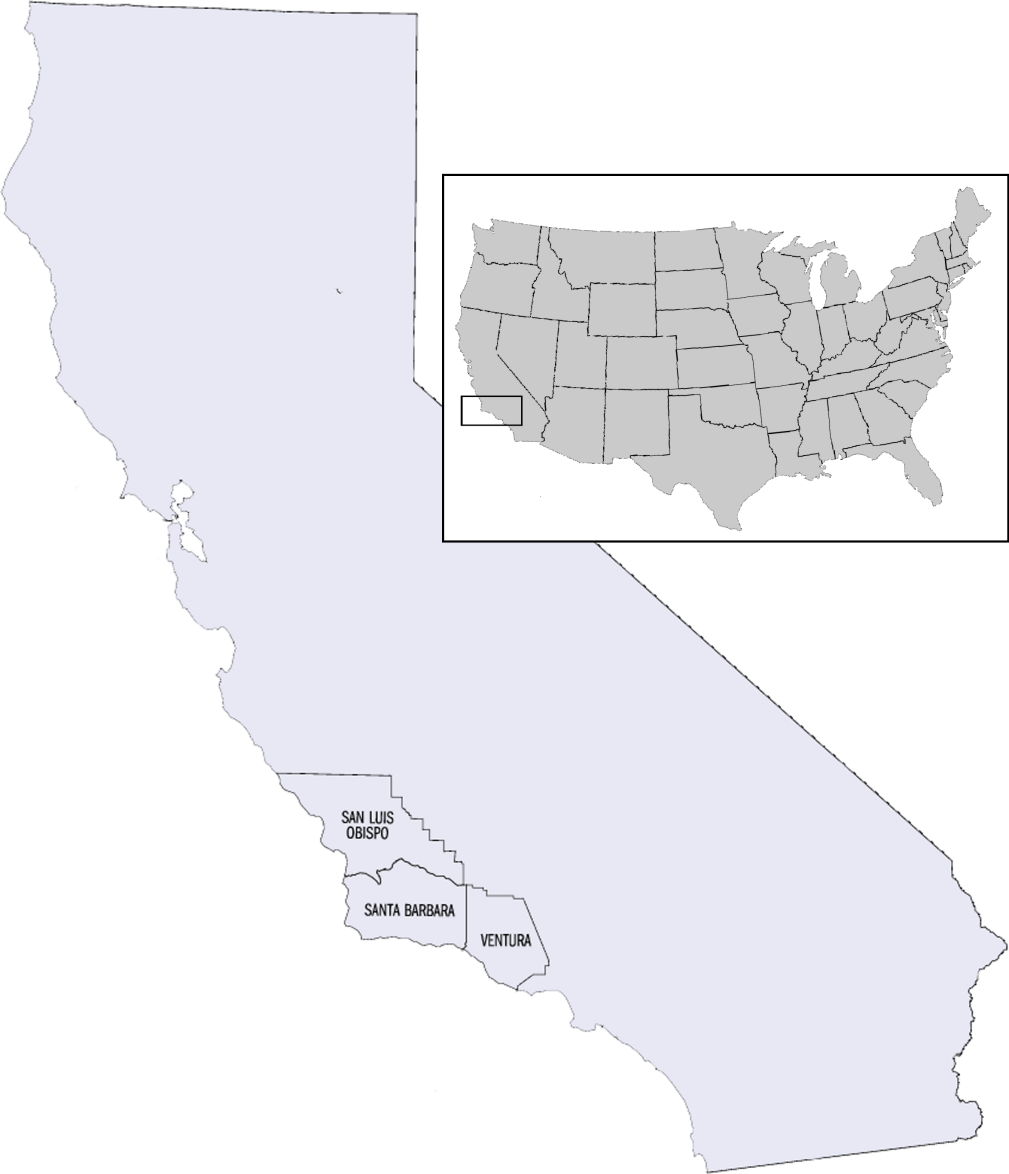
Map of study area depicting the Tri-County Area within California, United States of America. The Tri-County Area includes the counties of San Luis Obispo, Santa Barbara, and Ventura.

First our study evaluated the effect of SLR on 88 rare, largely endemic, coastal plant species within three counties (Tri-County Area) of California, USA (Fig. 2) by the end of this century. We then developed an SLR risk analysis model to evaluate the relationship between a plant’s characteristics and its likelihood of exposure to SLR in the future. In order to explore the effect of climate change on the current and future habitats of our plant species, we used MaxEnt (Phillips, Anderson & Schapire, 2006) to project species’ distributions under current and future climate conditions based on associations between bioclimatic/edaphic variables and current species locations. We then compared current and future suitable habitat area to the relative impact of SLR.

## Materials and Methods

### Species occurrence data

Using the CalFlora Plant Database available from The CalFlora Database (http://www.calflora.org), we selected 88 species in the Tri-County Area (an area of 23,948 km^2^) of California, USA (Fig. 2) that were likely candidates for exposure to SLR, given their occurrence at low elevations (0–30 m) and represented in our study a variety of different taxonomic families, habitat types, life histories, and status (CalFlora, 2014). There were other species that also existed below our elevation criteria but were not chosen for a variety of reasons such as lack of data, limited distribution etc. The selected 88 species represent 31 different taxonomic families; 6 habitat types including coastal fresh and brackish marshes, coastal dunes, scrub, coastal bluffs, and meadows and grasslands; multiple life histories including annuals, herbs, succulents, woody, and deciduous shrubs; a variety of elevation ranges; and a mix of state and federally listed species, as well as unlisted but rare species (Table S1).

Species occurrence data were extracted from the ‘RareFind’ dataset of the California Natural Diversity Database (CNDDB) (http://www.dfg.ca.gov/biogeodata/cnddb/). The CNDDB maintains information about the natural history and locations of rare, threatened, endangered, and special status species and natural communities of California and has been used for a variety of species distribution models (Hernandez et al., 2006; Williams et al., 2009; Regan et al., 2012). In CNDDB, location data for a species takes the form of polygonal occurrences, which are a rough proxy for populations. An occurrence is defined as the area of a cluster of individuals within 1/4 mile of one another and separated by at least that distance from other occurrences. We excluded all occurrences recorded before 1970 and any that were greater than 4 km in diameter in order to minimize outdated and uncertain values. Due to incomplete and unknown data on a number of individuals present within each occurrence, we assumed that populations were distributed evenly across occurrences. Thus, we included occurrences regardless of the number of individuals or clusters of populations known to be extant within them. The 88 species accounted for a total of 1091 occurrences used in our analyses.

### SLR projections

The SLR scenarios in this study were generated as part of the California Climate Impact Assessments which were produced from a downscaled global climate model (GCM) analyzed by the Scripps Institution of Oceanography (Cayan et al., 2009). The “high scenario” was a 1.4 m rise by 2100, while the “low” scenario was a 1.0 m rise by 2100 (Cayan et al., 2009). The coastal hazards of erosion and flooding associated with the impacts of the GCM outputs were projected for a variety of planning horizons using a total water level (tides + wave run-up) methodology (Revell et al., 2011). Coastal erosion model projections mapped all of San Luis Obispo County and most of Santa Barbara County, while the coastal flood extents were projected and mapped for the entire state of California. These projections of future coastal hazards were made available by the Pacific Institute, which conducted an initial statewide vulnerability assessment identifying critical infrastructure, habitats, and social demographics at risk from SLR (Heberger et al., 2011).

For coastal flooding, the mapped hazard extent was extrapolated from existing FEMA 100-year coastal Base Flood Elevations (BFEs), escalated by the projected amount of sea level rise. A 100-year flood is defined as a flood extent that has a 1% chance of being equaled or exceeded in a given year (FEMA, 2005). These BFEs, which calculated a maximum elevation of wave run-up at the shoreline, were mapped inland using a simple bathtub approach (FEMA, 2005). This approach likely overestimates the inland extent of coastal flooding, but in areas of combined fluvial and coastal flooding, may suitably represent the joint probability of a combined fluvial and coastal storm event (Revell et al., 2011). The coastal erosion hazards contained 3 components in the projected outputs: the effects of shoreline transgression from SLR, historic trends in shoreline change which provided an indirect accounting of sediment budget considerations, and the impact on erosion of a 100-year storm wave event (Revell et al., 2011). Inundation was mapped as the current extent of Mean High Water elevated by the SLR scenario over time by using a bathtub approach and ignoring hydraulic connectivity (Heberger et al., 2011). The resulting projections took the form of four general types of SLR related-threats (inundation, flooding, and cliff and dune erosion).

### SLR threat analysis

In order to analyze the threat of SLR to each species, the occurrences for the 88 species were combined with the above four SLR threat layers for the year 2100, including inundation, flooding, and cliff and dune erosion in the Tri-County Area. We compared the geographic area of the occurrence data with the geographic area of the SLR threat layers to determine the area of overlap. We used the area of overlap to calculate the percent of each occurrence exposed to SLR for each species. We examined the area of exposure by aggregating the geographic areas of the four SLR-related threats to determine where any threat might occur.

### SLR risk analysis

In order to determine the best predictors of exposure to SLR for our 88 species, we gathered a variety of physical, spatial, and biological plant characteristics related to each species, including life history, federal and California listing status, as well as each occurrence’s area, elevation, and distance from the coast (see Table S1). These variables included both continuous (e.g., elevation, distance) and categorical (e.g., life history, listing status) data. The continuous variables all had occurrence-level specificity, whereas the categorical variables only had species-level specificity. We ran multiple logistic regressions using R 2.15.1 (R Development Core Team, 2012), to determine which variables (including interactions) resulted in the best predictive models for exposure to SLR. We selected the best model based on two measures: the lowest Akaike Information Criterion (AIC) value (Akaike, 1973; Bozdogan, 1987) and statistically significant coefficients.

### Species distribution modeling

We modeled current and future habitat suitability using MaxEnt version 3.3.3k (Phillips, Anderson & Schapire, 2006), a machine-learning technique often used to model the spatial distribution of a species using environmental variables and species’ occurrence data (Gogol-Prokurat, 2011). Our species provides presence-only data. Although many SDMs require both presence and absence data to predict distributions, MaxEnt has been recognized to be particularly effective with presence only data (Phillips, Anderson & Schapire, 2006; Regan et al., 2012). Moreover, MaxEnt can partially compensate for incomplete and small data sets on species occurrence and perform with nearly maximal accuracy level under these conditions (Hernandez et al., 2006). This is ideal for rare species that typically have small populations.

Based on the results of the SLR Risk Analysis, we identified the 10 species that were most likely to be substantially impacted by SLR in the Tri-County Area. These were *Centromadia parryi* ssp. *australis, Chloropyron maritimum* ssp. *maritimum, Cirsium rhothophilum, Dithyrea maritima, Erigeron blochmaniae, Lasthenia glabrata* ssp. *coulteri, Monardella crispa, Monardella frutescens, Scrophularia atrata*, and *Suaeda californica*. We examined the effect of climate change on each species by modeling current and future habitat suitability in MaxEnt, based on current location data calculated from centroid of species occurrence polygons in California and six environmental inputs consisting of four bioclimatic (i.e., Mean Diurnal Range; Annual Precipitation; Precipitation in the Wettest Quarter; Growing degree days above 5 C) and two edaphic variables (i.e., Soil pH; and Available Water Holding Capacity). These environmental inputs have been used previously to model plant species distributions (Fitzpatrick et al., 2008; Riordan & Rundel, 2009; O’Donnell et al., 2012; Sheppard, 2013) because these variables were general factors influencing the distribution of a wide range of plant taxa (Woodward, 1987). The inclusion of soil characteristics has also been known to improve SDM performance when assessing climate change impacts (Austin & Van Niel, 2011) and has been used in various SDM studies (Syphard & Franklin, 2009; Regan et al., 2012; Belgacem & Louhaichi, 2013; Conlisk et al., 2013).

Historical climate was obtained from the Parameter-Elevation Regressions on Independent Slopes Model (PRISM) at Oregon State University, a method for extrapolating the measured historical data (Daly et al., 2002). Due to the large variability in long-range climatic predictions for 2100, we selected two GCMs: the Parallel Climate Model (PCM) (Washington et al., 2000) and the Geophysical Fluid Dynamics Lab (GFDL) (Delworth et al., 2006; Knutson et al., 2006) model, both used by the State of California for assessing climate change impacts because they produce accurate simulations of California’s recent historical climate but show different levels of sensitivity to greenhouse gas forcing (Cayan et al., 2008b). As all GCMs, GFDL and PCM project warmer conditions for southern California by the end of the 21st century, but PCM projects a more modest annual temperature increase (2.5 °C for PCM vs. 4.4 °C for GFDL) and winter precipitation change (+8% for PCM vs. −26% for GFDL) while the GFDL projects a generally drier future based on the IPCC’s A2 emissions scenario (i.e., business-as-usual) (Regan et al., 2012). We used downscaled monthly climate data from the two GCMs and PRISM (historical climate) at a grid size of 90-m resolution (Flint & Flint, 2012), and then calculated bioclimatic parameters based on the methods described in Sork et al. (2010) for Growing Degree Days and used the WORLDCLIM database (www.worldclim.org) for other bioclimatic parameters. The time horizon for this data is centered on 2085, as opposed to 2100, though it represents an end-of-century 30-year average with 2085 being the median (Flint & Flint, 2012).

We calibrated the MaxEnt model using the default value settings suggested by Phillips, Anderson & Schapire (2006). We set the random test percentage to 33%, which retains a percentage of the occurrences at random in order to evaluate the model and the rest of the occurrences were used to build the final models. We ran 10 replicate runs and averaged the results. We evaluated our models under the current climate by using the area underneath the receiver operating curve statistic (AUC) (Phillips, Anderson & Schapire, 2006). The AUC produces a single number between 0 and 1, where a higher AUC indicates a better model fit (Fielding & Bell, 1997; Giannini et al., 2012).

MaxEnt outputs are continuous probability layers for species occurrence under: (i) the historical climate with the PRISM climate model; and (ii) the two future projected climates with PCM and GFDL climate models. We converted the continuous probability maps from MaxEnt into binary presence/absence layers using a threshold value that minimizes the sum of sensitivity and specificity of the model (Jiménez-Valverde & Lobo, 2006). We removed current urban areas, which we deemed as unsuitable areas, from each of the three binary layers. However, these urban areas may not have included specific features such as seawalls that might have a direct impact on the future suitability of habitats. We then calculated the area of presence data to compare the relative gain or loss in habitat between the current and future scenarios allowing us to quantitatively compare the habitat change from impacts of climate change with SLR.

### Evaluating relative impacts of SLR and climate change

**Figure 3:**
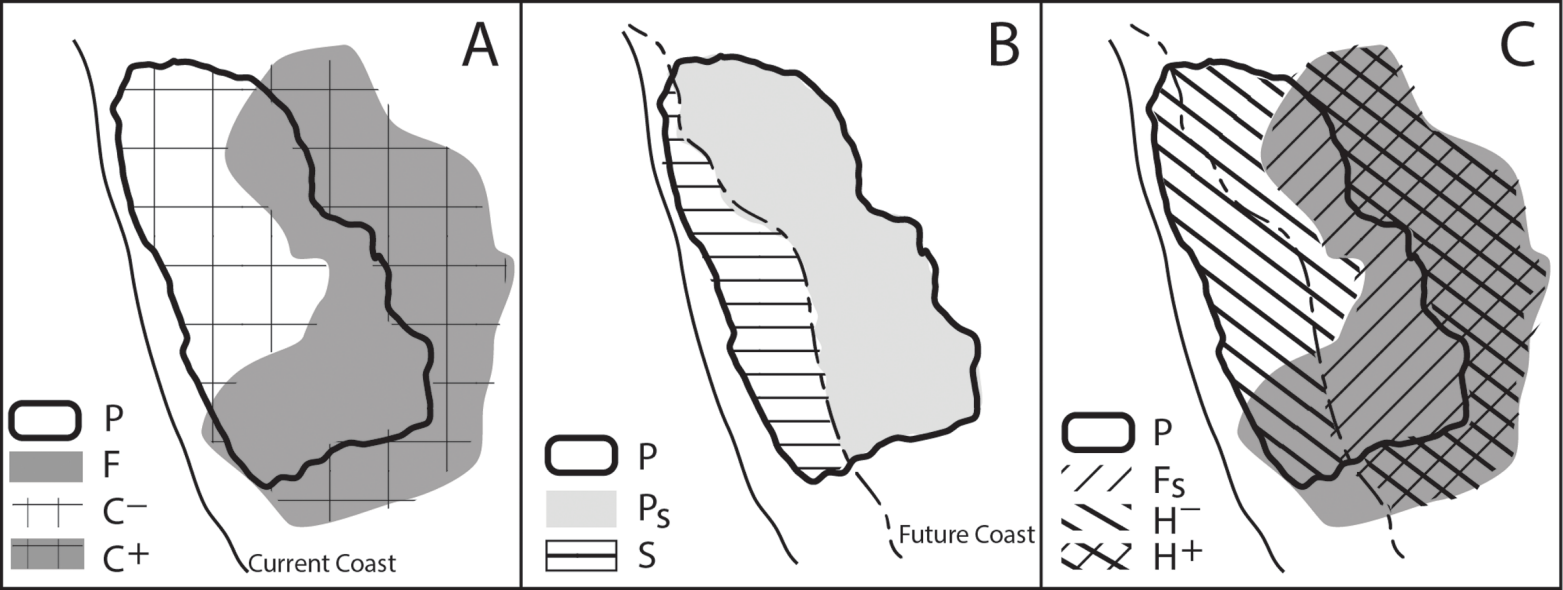
Conceptual model of the change in suitable habitat from the effects of SLR and climate change. In A, *P* represents the projected current suitable habitat and *F* represents the projected future suitable habitat. The area where there is no overlap between the two represents *C*, the net change in habitat due to climate change where *C*^+^ represents a gain in habitat while *C*^−^ represents a loss in habitat. In B, SLR overlaps the current suitable habitat (*P*), where *P_s_* is the remaining suitable habitat and *S* is the loss in suitable habitat resulting from SLR. In C, combining the effects of SLR and climate change (i.e., A and B), *F_s_* is the future suitable habitat that is not lost to SLR and *H* is the total change in suitable habitat. *H*^+^ represents a gain in suitable habitat (resulting from climate change) and *H*^−^ represents a loss in habitat (resulting from SLR and climate change).

Using the GIS layers of modeled current and future suitable habitat, along with the layer of projected SLR impact, we calculated several intermediate variables to quantify the relative effects of, and interactions between, SLR and climate change in determining changes in potential habitat for each species. Figure 3 illustrates a conceptual model of how the change in suitable habitat was affected by SLR and climate change. The direct effect of climate changes in air temperature and precipitation (*C*) is the change in suitable habitat area disregarding SLR, estimated as the difference in areas between the projected suitable habitat layer under one of the future climate projections (*F*) and that for historical climate (*P*) (Fig. 3A):

(1) \begin{eqnarray*} \displaystyle C=F-P.&&\displaystyle \end{eqnarray*}

Specifically the area projected to be lost from the current suitable habitat is identified as *C*^−^ and the area gained from future suitable habitat is *C*^+^.

The direct impact of SLR (*S*) is the reduction in current suitable habitat area caused by SLR:

(2) \begin{eqnarray*} \displaystyle S={P}_{S}-P,&&\displaystyle \end{eqnarray*}

where *P_s_* is the portion of the projected habitat layer based on the historical climate that does not overlap projected SLR (Fig. 3B). The total change in suitable habitat area (*H*) combines the effects of SLR and climate change:

(3) \begin{eqnarray*} \displaystyle H={F}_{S}-P,&&\displaystyle \end{eqnarray*}

where *F_s_* is the portion of the projected habitat layer based on future climate that does not overlap projected SLR (Fig. 3C). To further specify the direction of loss or gain in the change in habitat, *H*^+^ represents a gain in habitat while *H*^−^ represents the loss in habitat.

We write the combined impact of climate change and SLR as contributions from their direct effects and any spatial interactions (*I*) between the two:

(4) \begin{eqnarray*} \displaystyle H=C+S+I,&&\displaystyle \end{eqnarray*}

where

(5) \begin{eqnarray*} \displaystyle I=H-(C+S).&&\displaystyle \end{eqnarray*}

This interaction can be positive (Fig. 1A), negative (Fig. 1B) or zero (Fig. 1C).

We also calculated the proportional impact of SLR on habitat area under the future climate, *A*:

(6) \begin{eqnarray*} \displaystyle A=1-\left(\frac{{F}_{S}}{F}\right).&&\displaystyle \end{eqnarray*}

## Results

### SLR effects on current occurrences

**Figure 4:**
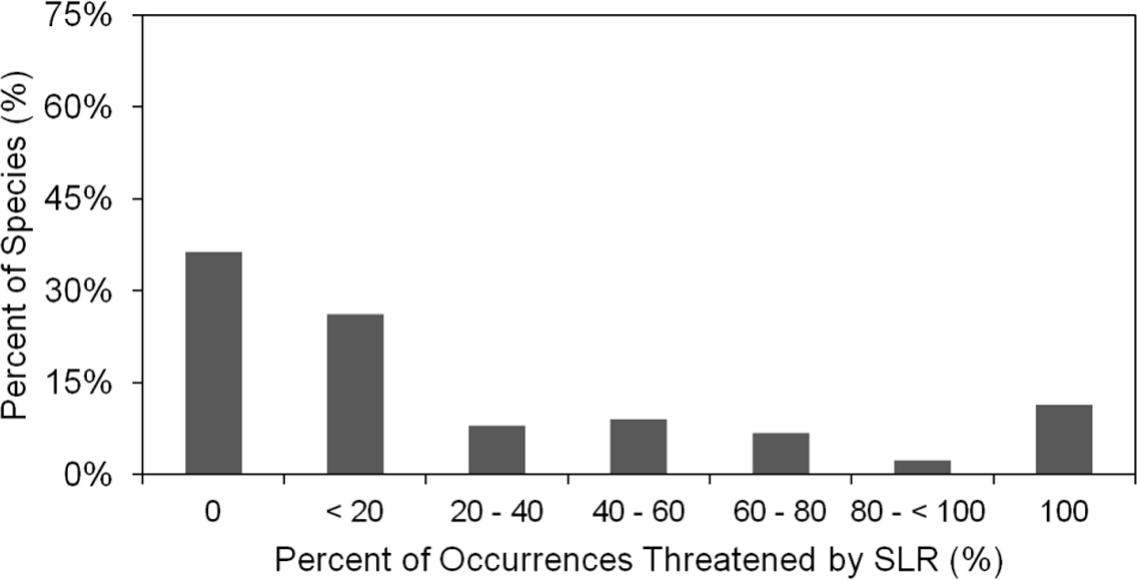
Histogram of percent of the 1,091 species, occurrences threatened by SLR by percent of species. This indicates the extent of threat for each species and the cumulative threat to all species.

**Figure 5:**
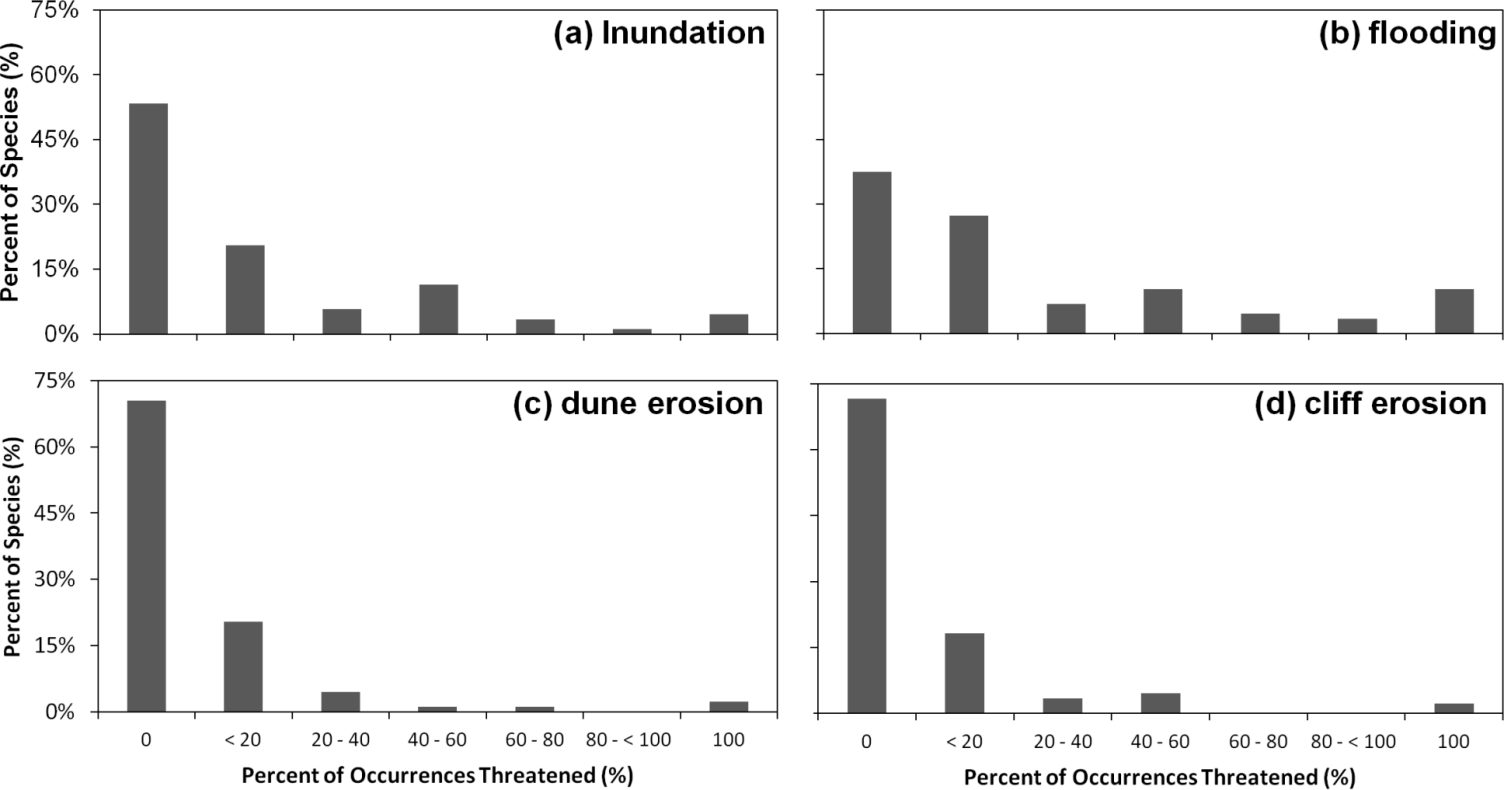
Histograms of percent of 1,091 species’ occurrences threatened by particular sea level rise threats. Each panel represents the histograms of each particular threat, (A) inundation, (B) flooding, (C) dune erosion, and (D) cliff erosion by percent of species.

We found that under the SLR projections for the year 2100, 17% of the 1091 occurrences of all species in our analysis would be affected by SLR, with a total of 10.6% threatened by routine inundation, 15.6% by a 100-year coastal flood, 5.9% by dune erosion, and 4.6% by cliff erosion. On the species level, we found that 65% of the 88 studied species are projected to have at least one occurrence impacted by SLR, with 12% of species having all of their occurrences within the SLR hazard zones (Fig. 4). However, nearly two thirds (63%) of the species are projected to have less than 20% of their occurrences at risk. The risk profile of the remaining species is fairly uniformly distributed between 20% and 100% (Fig. 4). Among all SLR threats, the threat profile from flooding alone closely mirrors the aggregate SLR threat profile. By contrast, inundation, dune erosion, and cliff erosion, are projected to affect almost 50% of species, with less than 5% of the species having all occurrences in the hazard zone (Fig. 5).

### SLR risk as a function of elevation and distance

**Figure 6:**
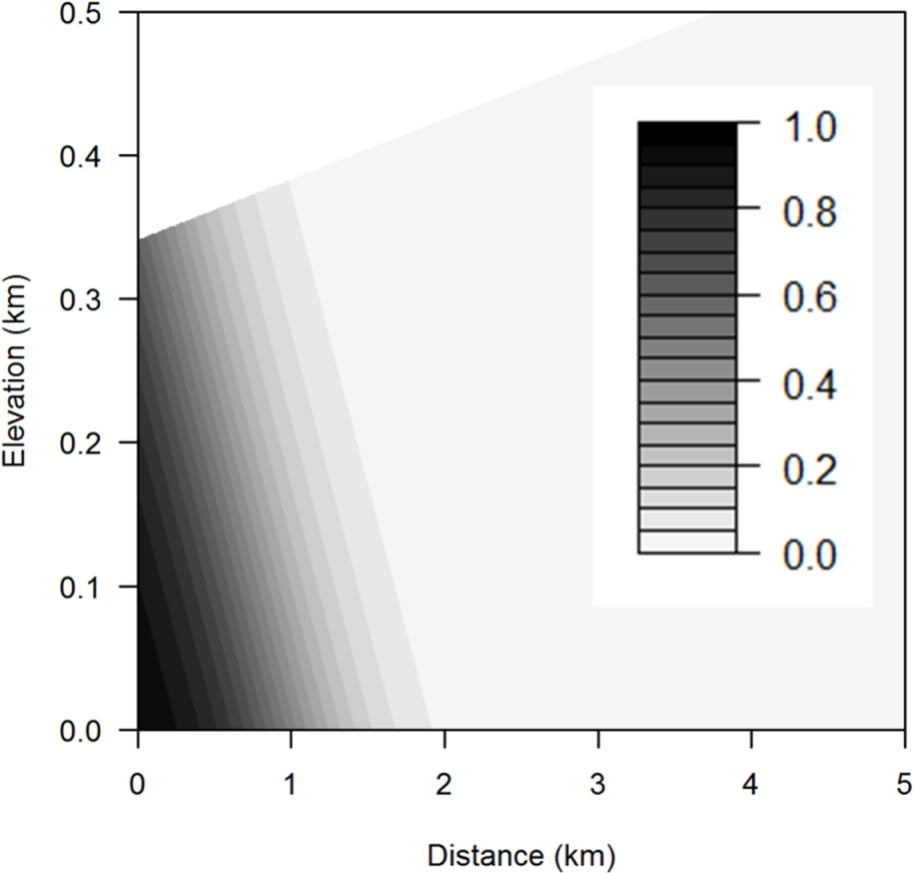
Aggregated sea level rise threats contour plot. Contour plot showing probability of exposure to aggregated sea level rise threats for any combination of elevation and distance from the coast using the mean occurrence area. The darker the area, the greater the probability of threat.

The best-fitted logistic regression model to explain the SLR exposure of species occurrences incorporated occurrence area, elevation, and distance from the coast (Table 1). None of the species-level variables (life history and listing status) were significant predictors of exposure to SLR (Table S1). Adding interaction terms did not improve the model. SLR threat to a species occurrence increases with occurrence area but decreases with elevation and distance from the coast (Table 1 and Fig. 6). Occurrences that are within 0.25 km of the coast and below 0.1 km in elevation are predicted to have a 100% chance of exposure to SLR.

**Figure 7:**
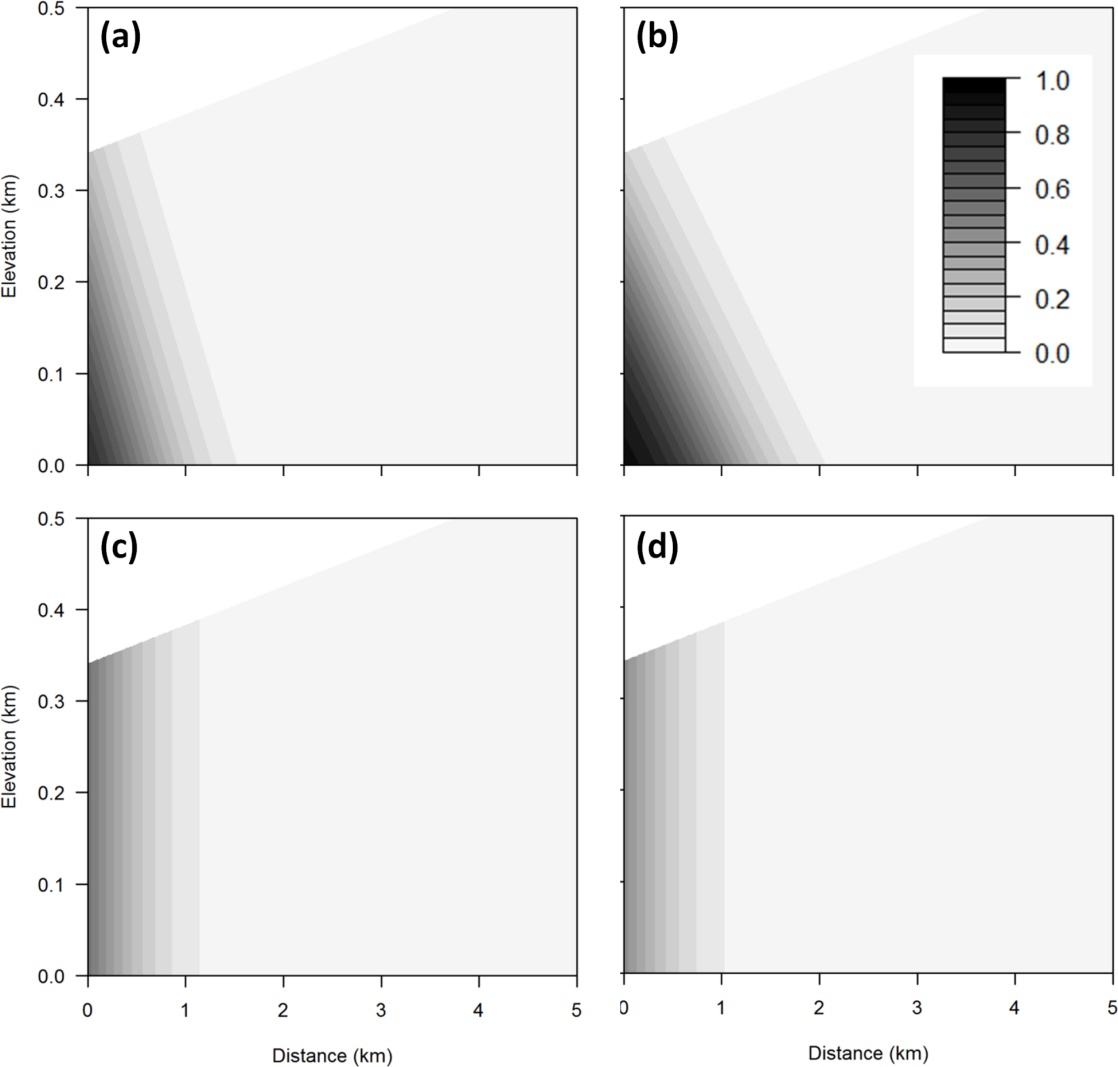
Sea level rise threats contour plot. Contour plot showing probability of exposure to sea level rise threats (A) inundation, (B) flooding, (C) dune erosion, and (D) cliff erosion for any combination of elevation and distance from the coast using a mean occurrence area. The darker the area, the greater the probability of threat.

The probability of exposure to inundation and flooding is qualitatively similar to that for the aggregate threat, with risk from flooding extending further inland than inundation (Table 2 and Fig. 7). In contrast, exposure to dune and cliff erosion depends only on distance from coast and occurrence area, but not elevation (Table 2 and Fig. 7).

**Table 1:** Coefficients table for Aggregate SLR risk model. Logistic regression for the probability that a given species occurrence will be affected by SLR. Terms with *P* > 0.05 have been dropped.

	Estimate	Std. error	*Z* value	*Pr*(>|*z*|)
(Intercept)	1.8792	0.2443	7.692	1.44e-14
Area (km^2^)	0.8787	0.1201	7.317	2.54e-13
Elevation (km)	−7.5795	3.2419	−2.388	0.0194
Distance (km)	−3.0909	0.3844	−8.041	8.88e-16

**Table 2:** Parameter estimates for inundation risk model. Logistic regression for the probability that a given species occurrence will be affected by each SLR threat component.

Parameter	Inundation	Flooding	Dune erosion	Cliff erosion
(Intercept)	0.5871^*^	1.5221^***^	−0.52014^*^	−0.88882^**^
Area (km^2^)	0.7189^***^	0.8693^***^	0.48797^***^	0.48498^***^
Elevation (km)	−7.9667^*^	−12.4263^**^		
Distance (km)	−2.9035^***^	−2.6919^***^	−2.65244^***^	−2.58650^***^

**Notes.**

* *P* < 0.05.

** *P* < 0.001.

*** *P* < 0.0001.

### Effects of climate change and SLR on habitat (species distribution modeling)

All runs for our 10 species produced consistently high AUC values greater than 0.95, indicating that MaxEnt modeled and predicted the current distribution of species effectively. Four species (*Cirsium rhothophilium, Erigeron blochmaniae, Monardella crispa, and Monardella frutescens*) were projected to have no habitat left in the study region under both the PCM and GFDL future climate models.

**Figure 8:**
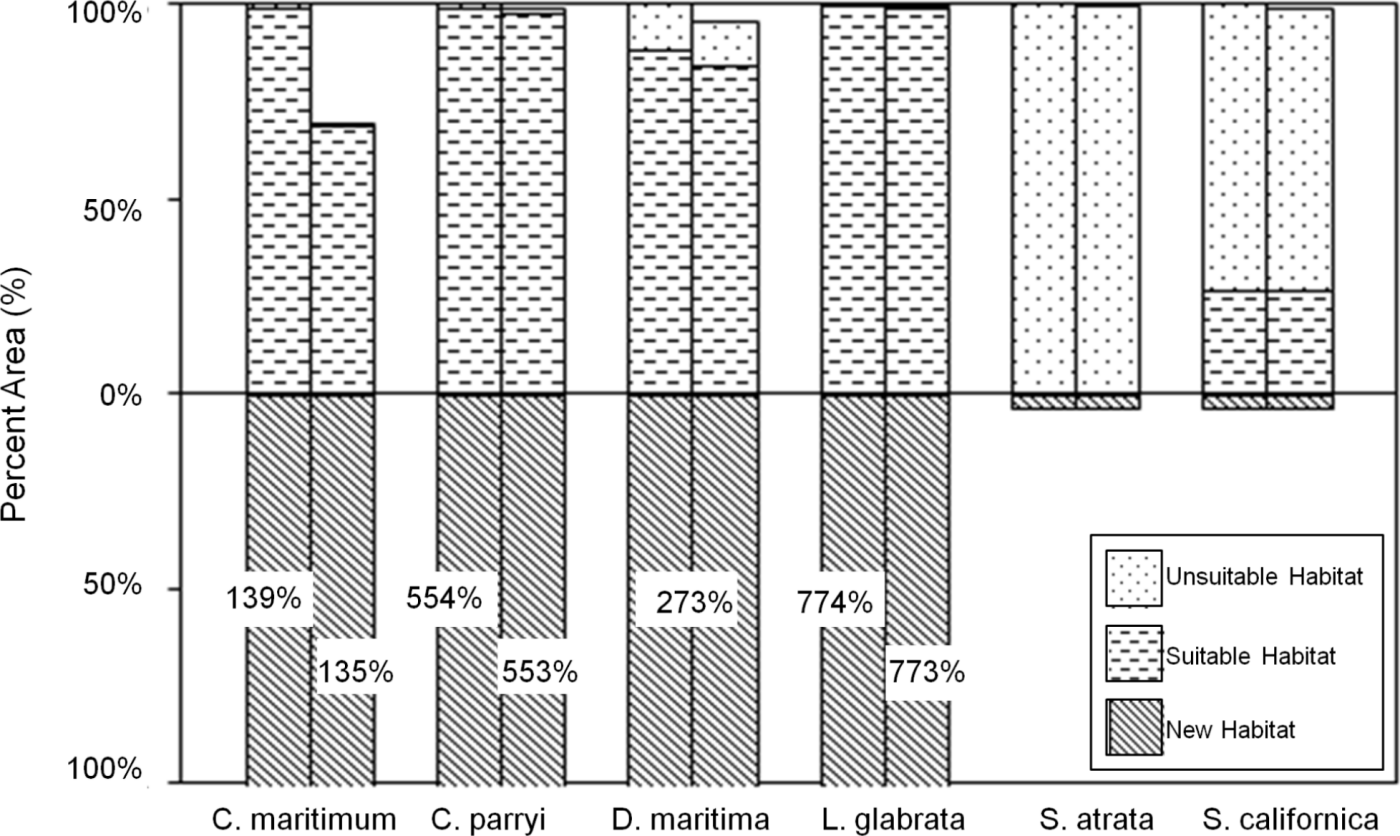
Current and future habitat projected by the GFDL climate model within the Tri-County Area, expressed as percent of current habitat. Current habitat is represented above the *x*-axis while future habitat is represented below the *x*-axis. Columns arising above or below the *x*-axis represent a gain in percent habitat. The first set of columns for each species indicates all areas within the Tri-County, so current habitat is 100% while future habitat is more than 100% (exceeding the graph) for some species. The second set of columns for each species indicates all areas within the Tri-County Area after loss to sea level rise. Unsuitable habitat is habitat that will become unsuitable in the future due to climate change. Suitable habitat is current habitat that will remain suitable even with climate change. New habitat is the same as the future habitat that will be created as a result of climate change.

Under the GFDL climate model, four species (*C. maritimum* ssp. *maritimum, C. parryi* ssp. *parryi, D. maritimum, and L. glabrata* ssp. *coulteri*) are projected to significantly expand habitats with minimal loss to current modeled habitat (Fig. 8). With SLR, only *C. maritimum* ssp. *maritimum* loses as much as 40% of the current habitat. *S. atrata* is projected to have only a very small amount of future suitable habitat, and this habitat does not overlap with the current habitat projected for this species. *S. californica* is projected to maintain about 25% of its current habitat under the GFDL model, with a very modest habitat expansion into new areas and no significant losses to SLR.

**Figure 9:**
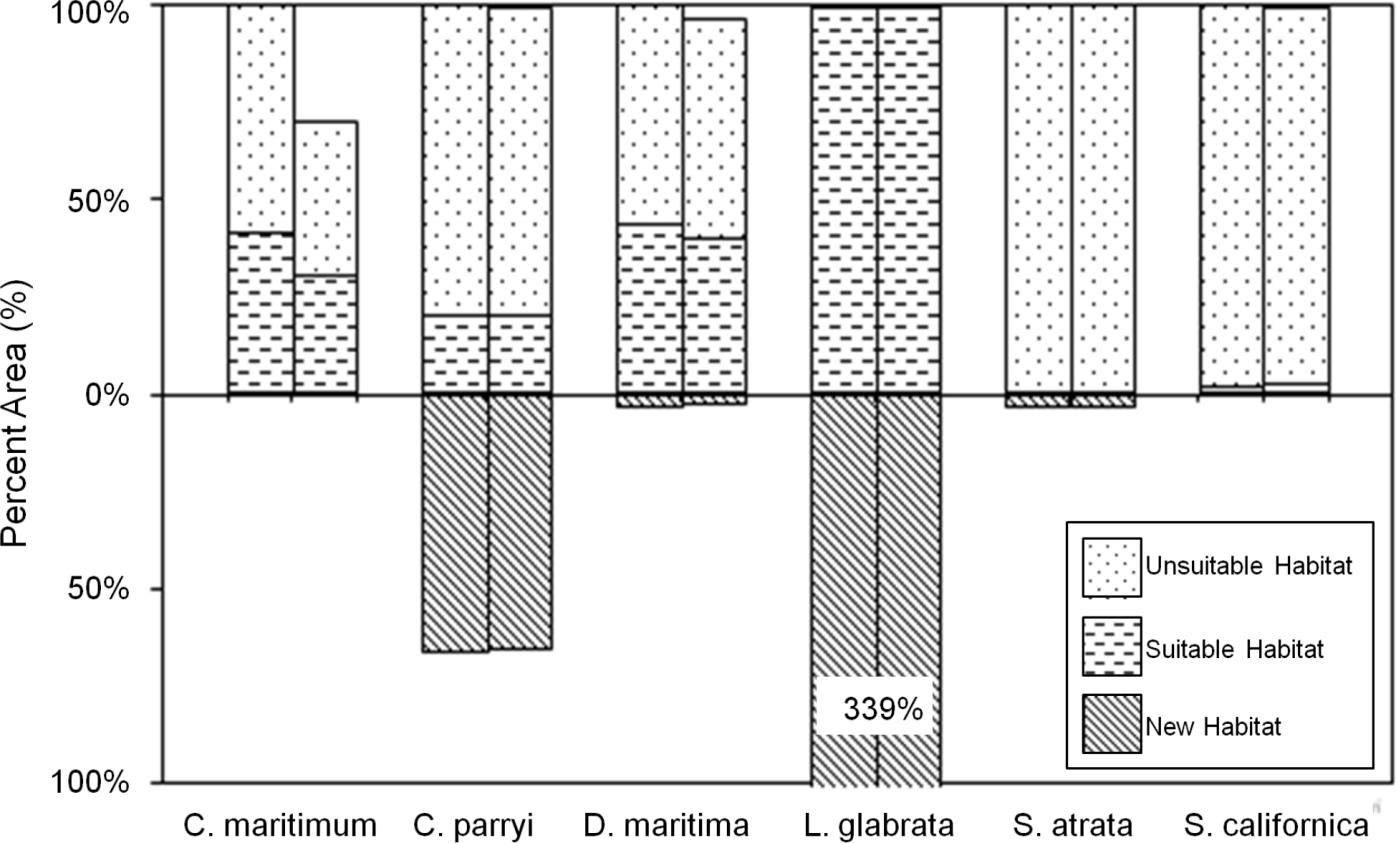
Current and future habitat projected by the PCM climate model within the Tri-County Area expressed as percent of current habitat. Current habitat is represented above the *x*-axis while future habitat is represented below the *x*-axis. Columns arising above or below the *x*-axis represent a gain in percent habitat. The first set of columns for each species indicates all areas within the Tri-County, so current habitat is 100% while future habitat is less than 100% for most species except one. The second set of columns for each species indicates all areas within the Tri-County Area after loss to sea level rise. Unsuitable habitat is habitat that will not be suitable in the future due to climate change. Suitable habitat is current habitat that will remain suitable even with climate change. New habitat is the same as the future habitat that will be created as a result of climate change.

The PCM climate model primarily projects a contraction in future habitat (Fig. 9). Only two species are projected to gain significant habitat under the PCM climate model; *L. glabrata* ssp. *coulteri* will gain extensive suitable habitat (+339% habitat relative to current habitat) and *C. parryi* ssp. *parryi* will gain some new suitable habitat (+65% habitat relative to current habitat) All species, except *L. glabrata* ssp. *coulteri*, maintain less than 45% of their current habitat under the PCM future climate model, with notable losses from SLR for *C. maritiumum* ssp. *maritimum* (Fig. 9).

The total loss of current habitat due to SLR is projected to be similar across species (Table 3). In contrast, the projected changes in habitat resulting from climate change are much more variable across species and climate models. In terms of the area of habitat lost, the impact of SLR can be as much as half the magnitude of the projected impact of climate change (*C. maritimum* under PCM), but is generally a much smaller component of future habitat change (as little as 0.1%). Comparing the percent area lost due to SLR for the current and future climate models reveals that the proportional impact of SLR is generally less in the future than at present (the exceptions are *D. maritima* and *S. californica* under the PCM climate model). Additionally, the interaction between SLR and climate change is on the same order of magnitude as the effect of SLR alone (Table 3).

**Table 3:** Changes in modeled habitat areas under climate change scenarios and projected sea level rise. Negative values indicate habitat contraction, whereas positive values indicate habitat expansion. Present habitat (P) is the total current habitat projected under the historical climate (PRISM). Total habitat change (H) is calculated as the present projected habitat subtracted from the future projected habitat under SLR. Habitat change due to climate change (C) was calculated as the present projected habitat subtracted from the future projected habitat without accounting for SLR. Habitat change due to SLR (S) was calculated as present projected habitat under SLR subtracted from present projected habitat. The percent area lost to SLR (A) is the percent of total suitable habitat that will be exposed to SLR.

Species	Present Habitat (P) (sq km)	Total Habitat Change (H) (sq km)		Habitat Change due to Climate Change (C) (sq km)		Habitat Change due to SLR (S) (sq km)	Interaction (I) (sq km)		Percent Area Lost to SLR (A) (%)		
	PRISM	PCM	GFDL	PCM	GFDL	PRISM	PCM	GFDL	PRISM	PCM	GFDL
*C. maritimum*	212.3	−14.7	+22.0	−12.2	+29.3	−6.5	4.0	−0.8	30.63	27.78	14.52
*C. parryi*	585.3	−83.0	+3, 222.2	−80.3	+3, 236.8	−7.1	4.3	−7.5	1.21	0.55	0.38
*D. maritime*	214.9	−123.9	+552.0	−114.9	+562.0	−9.2	0.2	−0.8	4.30	9.01	1.29
*L. glabrata*	1,265.5	+4, 271.8	+9, 777.7	+4, 283.4	+9, 795.5	−9.2	−2.3	−8.6	0.73	0.21	0.16
*S. atrata*	1,499.2	−1,439.2	−1,436.9	−1,439.2	−1,436.9	−6.1	6.1	6.1	0.40	0.00	0.00
*S. californica*	1,032.1	−1,008.7	−726.5	−1,007.3	−725.3	−8.7	7.4	7.6	0.85	5.31	0.37

## Discussion

Sea level rise and climate change could have significant impacts to rare plant species along the California, USA coast. To reiterate, our study addressed the following questions: (1) What is the extent of the impact of SLR on rare plant species along the central California, USA coast; (2) Which plant characteristics are the best predictors of exposure to SLR; (3) To what extent will climate change shift the current habitat of rare coastal plant species in the future; (4) What is the relative impact of climate change compared to SLR on the habitat of species? In order to investigate the effect of SLR, we identified species that could be at risk by comparing the overlap of their occurrences with the most recent projections of SLR-related threats (inundation, flooding, and cliff and dune erosion) for 2100. Our results indicate that SLR alone could cause the regional extinction (loss of all known occurrences) of over 12% of the species considered in this study (Fig. 4). Similar studies have predicted losses in wetland and marsh habitat that range from 2% to over 45% (Nicholls, Hoozemans & Marchand, 1999; Craft et al., 2009).

We also used a variety of plant characteristics including geographical parameters in our regression model to predict the SLR risks on each species and found that area, elevation, and distance from the coast are the best predictors of a particular species occurrence’s exposure to SLR. Having accounted for these geographical factors, no other species-level traits predicted SLR exposure. Thus, plant species that are closer to the coast, lower in elevation, and smaller in terms of their area of occurrence would be most likely to face exposure to SLR independent from species characteristics. In particular, species found at very low elevations have a high likelihood of exposure to SLR (Figs. 4 and 5). These species may face a high extinction risk without active management to improve their resilience.

Turning to the direct effects of climate, our results suggest that climate change may cause a substantial shift in suitable habitat for many rare coastal plant species by the end of the century (Figs. 8 and 9). While our two climate models projected different outcomes (the GFDL model projected larger habitat expansions and smaller habitat losses than the PCM model), the models produced qualitatively similar projections for 60% of the 10 species that we examined: four species had no future habitat within the Tri-County, two species had substantial habitat loss of 70%–97%, and one species experienced a substantial expansion in future habitat of up to 700%. Thus, at least half of the rare coastal plant species face regional threat from climate change. A European study found habitat loss due to climate change ranging between 2.3%–38.1% (Randin et al., 2009). Another study in the European Alps found that while 60% of plant species experienced low rates of habitat loss (<5%), the other 40% of species are likely to lose more than 90% of their suitable habitat (Theurillat & Guisan, 2001). Therefore, regardless of how climate may change in California, some rare species will be lost without appropriate preventative action.

Individually, we found the effects of SLR to be relatively similar across our species while climate change effects were orders of magnitude higher for certain species and far more variable. While SLR alone always constitutes a loss in habitat area, in some cases the absolute and proportional impacts of SLR on plant species can be affected by climate change. For some species the interactive effects mirrored the scenarios of our conceptual model of SLR impacts from suitable habitat shifts due to climate change (Fig. 1). When suitable habitat moves away from the coast under future climate, as with *S. atrata*, the result is a positive interaction between climate change and SLR (Fig. 1A), constituting an overall gain in habitat. When suitable habitat moves towards the coast under future climate, as with *G. glabrata*, the result is a negative interaction between climate change and SLR (Fig. 1B). When suitable habitat moves along the coastline, as with *D. maritimum*, the result is essentially a zero (or very small) interaction (Fig. 1C). In general, when taking SLR and climate change together, we found that the interaction was similar in scale to the effect of SLR alone.

As in most research projecting future species distributions, our study includes a number of critical assumptions. First, the SLR projections did not capture other abiotic interactions that may prove important factors in influencing future species distributions, such as fluvial flooding and in particular, salt-water intrusion into coastal aquifers and wetlands. While many coastal species have some degree of tolerance to saltwater, SLR will likely increase inundation rates, allowing saltwater to contaminate fresh ground and surface water stores, which could alter vegetation drastically (Heberger et al., 2009). Saltwater intrusion would likely expand the extent of our SLR models farther inland than predicted at an accelerating rate over time (Heberger et al., 2009).

In projecting future habitat ranges of species, SDMs have a number of limitations. SDMs do not typically account for limits to a species’ dispersal; they simply aim to predict the potential range of a species under a new climate. The ability of a species to migrate at a sufficient rate to keep pace with changing climate depends on the dispersal characteristics of that species (Collingham & Huntley, 2000). Plant species are far more limited in their dispersal capability than motile species, and rare plant species tend to be further limited (Graham & Grimm, 1990; Collingham, Hill & Huntley, 1996). Given the limited rate of dispersal for most plants, and the variability in suitable habitat (Figs. 8 and 9), the actual future range of most of our species may be far smaller than the projected future range.

As with any SDM, MaxEnt assumes that species will not exhibit phenotypic adaptation to new environmental conditions (Hoagland et al., 2011) or rapid evolutionary change in response to shifting climate conditions (Wiens et al., 2009). Given that we are studying rare and frequently sensitive species, these are reasonable assumptions. Further, MaxEnt assumes that the current distribution of a species encompasses its entire climatic range, which may not be the case for rare species with only a handful of occurrences. Lastly, MaxEnt does not account for certain inter-specific interactions, such as dependence on pollinators, competition with invasive species, and herbivory (Fitzpatrick et al., 2008). For example, the geographic and ecological distribution of the hemiparasitic *C. maritimum* is largely dependent on the distribution of its host plant as well as pollinators such as bees and flies (USFWS, 2009).

Our SDM random sampling area (background) included the entire state of California, which may have led to our model overestimating available suitable habitat, largely because dispersal to far-flung areas is unlikely. Given that our projections cover a relatively short time span, it is reasonable to assume that most species will have little ability to escape the effects of SLR and climate change through passive dispersal or evolution. Our model also may not have captured local adaptations or the effect of microhabitats. Along with abiotic environmental variables, other factors such as inter-species interactions, ecosystem dynamics, and land use changes could also influence species’ survival and colonization success. For example, promising research has begun to evaluate the ability of salt marsh species to migrate upslope, which could improve any future modeling efforts (Feagin et al., 2010; Wasson, Woolfolk & Fresquez, 2013).

For most rare species, we do not know which climatic and edaphic variables are most important for predicting suitable habitat (USFWS, 2009; USFWS, 2010). As such, there is a high level of uncertainty about which environmental inputs are appropriate for use in MaxEnt. It was not feasible to model the distributions of our 10 species using more tailored, species-specific sets of environmental variables, as data on habitat preferences for many rare species are not available. Future modeling efforts that select more species-specific environmental variables may provide more insights in projecting suitable habitat for particular species. It would also be useful to expand our selection to the 88 species as well as to currently non-coastal species that might become coastal as sea levels rise.

This research represents an important first step in assessing the emerging threats to coastal plant species by addressing the factors relating to SLR and climate change. The areas where we have identified future suitable habitat could theoretically be colonized but likely would need human assistance as the distances are too large for natural dispersal. Our research implies that there is a need for human-assisted migration or similar management approached to preserve species that are unlikely to survive the effects of SLR and climate change. Further study and proactive management are required to ensure the survival of coastal plant species against both the short- and long-term threats of SLR and climate change.

## Supplemental Information

10.7717/peerj.958/supp-1 Table S1 Species List used in sea level rise risk analysis Table shows each species and their characteristics. Each species also includes their total number of occurrences and how many are exposed to inundation, flooding, and cliff and dune erosion.
