# Supplementary material for: Impacts of sea level rise and climate change on coastal plant species in the central California coast
Source: PeerJ. 2015 May 12;3:e958. doi: 10.7717/peerj.958 (PMC4435450; doi:10.7717/peerj.958)
Supplement: Table S1 — Table shows each species and their characteristics. Each species also includes their total number of occurrences and how many are exposed to inundation, flooding, and cliff and dune erosion. [file peerj-03-958-s001.docx]

**Table S-1.** Species List used in sea level rise risk analysis.

| Name | Family | Life History | Habitat Type | Federal listed | State listed | Number of Occurrences | | | | |
| --- | --- | --- | --- | --- | --- | --- | --- | --- | --- | --- |
|  |  |  |  |  |  | Total | Threatened by Inundation | Threatened by Flooding | Threatened by Dune Erosion | Threatened by Cliff Erosion |
| *Agrostis hooveri* | Poaceae | Perennial Herb | Chaparral | None | None | 21 | 0 | 0 | 0 | 0 |
| *Allium hickmanii* | Alliacea | Perennial Herb | Prairie | None | None | 8 | 0 | 2 | 0 | 0 |
| *Aphanisma blitoides* | Chenopodiaceae | Annual Herb | Coastal Sage Scrub | None | None | 2 | 0 | 0 | 0 | 1 |
| *Arctostaphylos cruzensis* | Ericaceae | Perennial Shrub | Chaparral | None | None | 14 | 0 | 1 | 0 | 0 |
| *Arctostaphylos hookeri ssp. hearstiorum* | Ericaceae | Perennial Tree/Shrub | Salt Marsh | None | Endangered | 5 | 0 | 0 | 0 | 0 |
| *Arctostaphylos morroensis* | Ericaceae | Perennial Shrub | Coastal Strand | Threatened | None | 7 | 2 | 3 | 2 | 1 |
| *Arctostaphylos pechoensis* | Ericaceae | Perennial Shrub | Chaparral | None | None | 12 | 0 | 0 | 0 | 0 |
| *Arctostaphylos purissima* | Ericaceae | Perennial Shrub | Chaparral | None | None | 9 | 0 | 0 | 0 | 0 |
| *Arctostaphylos rudis* | Ericaceae | Perennial Shrub | Chaparral | None | None | 21 | 1 | 1 | 0 | 1 |
| *Arenaria paludicola* | Caryophyllaceae | Perennial Herb | Freshwater Marsh | Endangered | Endangered | 11 | 1 | 1 | 1 | 0 |
| *Astragalus didymocarpus var. milesianus* | Fabaceae | Annual Forb | Coastal Sage Scrub | None | None | 11 | 2 | 2 | 1 | 1 |
| *Astragalus pycnostachyus var. lanosissimus* | Fabaceae | Perennial Herb | Salt Marsh | Endangered | Endangered | 3 | 2 | 2 | 0 | 0 |
| *Atriplex coulteri* | Chenopodiaceae | Perennial Herb | Coastal Strand | None | None | 6 | 3 | 5 | 1 | 3 |
| *Atriplex serenana var. davidsonii* | Chenopodiaceae | Annual Herb | Coastal Sage Scrub | None | None | 6 | 0 | 3 | 1 | 2 |
| *Baccharis plummerae ssp. glabrata* | Asteraceae | Perennial Shrub | Coastal Sage Scrub | None | None | 2 | 1 | 1 | 0 | 0 |
| *Bloomeria humilis* | Themidaceae | Perennial Herb | Chaparral | None | Rare | 2 | 1 | 1 | 0 | 0 |
| *Calochortus clavatus var. recurvifolius* | Liliaceae | Perennial Herb | Chaparral | None | None | 5 | 1 | 1 | 0 | 0 |
| *Calochortus obispoensis* | Liliaceae | Perennial Herb | Chaparral | None | None | 31 | 0 | 0 | 0 | 0 |
| *Calochortus simulans* | Liliaceae | Perennial Herb | Chaparral | None | None | 35 | 0 | 0 | 0 | 0 |
| *Calystegia sepium ssp. binghamiae* | Convolvulaceae | Perennial Herb | Salt Marsh | None | None | 1 | 0 | 1 | 0 | 0 |
| *Calystegia subacaulis ssp. episcopalis* | Convolvulaceae | Perennial Herb | Chaparral | None | None | 25 | 0 | 2 | 0 | 0 |
| *Camissonia hardhamiae* | Onagraceae | Annual Herb | Chaparral | None | None | 12 | 0 | 0 | 0 | 0 |
| *Carex obispoensis* | Cyperaceae | Perennial Herb | Prairie | None | None | 23 | 1 | 3 | 0 | 0 |
| *Castilleja ambigua ssp. insalutata* | Orobanchaceae | Annual Herb | Marsh | None | None | 4 | 2 | 3 | 0 | 0 |
| *Castilleja densiflora ssp. obispoensis* | Orobanchaceae | Annual herb | Grassland | None | None | 42 | 5 | 7 | 0 | 0 |
| *Ceanothus hearstiorum* | Rhamnaceae | Perennial Shrub | Prairie | None | Rare | 6 | 0 | 0 | 0 | 0 |
| *Ceanothus maritimus* | Rhamnaceae | Perennial Shrub | Chaparral | None | Rare | 4 | 1 | 1 | 0 | 0 |
| *Centromadia parryi ssp. australis* | Asteraceae | Annual Herb | Marsh | None | None | 11 | 1 | 6 | 2 | 3 |
| *Centromadia parryi ssp. congdonii* | Asteraceae | Annual Herb | Grassland | None | None | 12 | 0 | 0 | 0 | 0 |
| *Centromadia parryi ssp. parryi* | Asteraceae | Annual Herb | Chaparral | None | None | 1 | 0 | 0 | 0 | 0 |
| *Chloropyron maritimum ssp. maritimum* | Orobanchaceae | Annual Herb | Coastal Strand | Endangered | Endangered | 13 | 12 | 12 | 1 | 0 |
| *Chorizanthe breweri* | Polygonaceae | Annual Herb | Chaparral | None | None | 30 | 1 | 1 | 1 | 0 |
| *Chorizanthe pungens var. pungens* | Polygonaceae | Annual Herb | Coastal Strand | Threatened | None | 1 | 1 | 1 | 0 | 0 |
| *Chorizanthe rectispina* | Polygonaceae | Annual Herb | Chaparral | None | None | 21 | 0 | 0 | 0 | 0 |
| *Cirsium fontinale var. obispoense* | Asteraceae | Perennial Herb | Chaparral | Endangered | Endangered | 13 | 0 | 0 | 0 | 0 |
| *Cirsium occidentale var. compactum* | Asteraceae | Perennial Herb | Coastal Strand | None | None | 12 | 7 | 7 | 0 | 2 |
| *Cirsium rhothophilum* | Asteraceae | Perennial Herb | Coastal Strand | None | Threatened | 24 | 19 | 21 | 13 | 12 |
| *Cirsium scariosum var. loncholepis* | Asteraceae | Perennial Herb | Coastal Strand | Endangered | Threatened | 19 | 3 | 3 | 2 | 0 |
| *Cladium californicum* | Cyperaceae | Perennial Herb | Freshwater Marsh | None | None | 3 | 0 | 0 | 0 | 0 |
| *Clarkia speciosa ssp. immaculata* | Onagraceae | Annual Herb | Chaparral | Endangered | Rare | 19 | 0 | 0 | 0 | 0 |
| *Cordylanthus rigidus ssp. littoralis* | Orobanchaceae | Annual Herb | Coastal Strand | None | Endangered | 21 | 0 | 0 | 0 | 0 |
| *Deinandra increscens ssp. villosa* | Asteraceae | Annual Herb | Coastal Bluffs | Endangered | Endangered | 49 | 0 | 3 | 0 | 2 |
| *Delphinium parryi ssp. blochmaniae* | Ranunculaceae | Perennial Herb | Coastal Strand | None | None | 15 | 0 | 0 | 0 | 0 |
| *Dithyrea maritima* | Boraginaceae | Perennial Herb | Coastal Strand | None | Threatened | 14 | 8 | 9 | 10 | 1 |
| *Dudleya abramsii ssp. bettinae* | Crassulaceae | Perennial Herb | Chaparral | None | None | 8 | 0 | 1 | 0 | 0 |
| *Dudleya abramsii ssp. murina* | Crassulaceae | Perennial Herb | Chaparral | None | None | 27 | 0 | 0 | 0 | 0 |
| *Dudleya blochmaniae ssp. blochmaniae* | Crassulaceae | Perennial Herb | Grassland | None | None | 21 | 1 | 1 | 1 | 1 |
| *Dudleya parva* | Crassulaceae | Perennial Herb | Coastal Sage Scrub | Threatened | None | 11 | 0 | 0 | 0 | 0 |
| *Dudleya verityi* | Crassulaceae | Perennial Herb | Chaparral | Threatened | None | 3 | 0 | 0 | 0 | 0 |
| *Erigeron blochmaniae* | Asteraceae | Perennial Herb | Coastal Strand | None | None | 23 | 5 | 8 | 7 | 2 |
| *Eriodictyon altissimum* | Boraginaceae | Perennial Shrub | Chaparral | Endangered | Endangered | 6 | 0 | 0 | 0 | 0 |
| *Eriodictyon capitatum* | Boraginaceae | Perennial Shrub | Chaparral | Endangered | Rare | 6 | 0 | 0 | 0 | 0 |
| *Eriogonum crocatum* | Polygonaceae | Perennial Herb | Chaparral | None | Rare | 13 | 0 | 0 | 0 | 0 |
| *Eryngium aristulatum var. hooveri* | Apiaceae | Both Herb | Freshwater Marsh | None | None | 3 | 1 | 1 | 0 | 1 |
| *Galium californicum ssp. luciense* | Rubiaceae | Perennial Herb | Forest | None | None | 1 | 1 | 1 | 0 | 1 |
| *Horkelia cuneata ssp. puberula* | Rosaceae | Perennial Herb | Chaparral | None | None | 22 | 0 | 0 | 0 | 0 |
| *Horkelia cuneata ssp. sericea* | Rosaceae | Perennial Herb | Northern Coastal Scrub | None | None | 8 | 0 | 1 | 0 | 0 |
| *Lasthenia californica ssp. macrantha* | Asteraceae | Annual Herb | Northern Coastal Scrub | None | None | 2 | 2 | 2 | 0 | 0 |
| *Lasthenia conjugens* | Asteraceae | Annual Herb | Grassland | Endangered | None | 1 | 0 | 1 | 1 | 1 |
| *Lasthenia glabrata ssp. coulteri* | Asteraceae | Annual Herb | Salt Marsh | None | None | 12 | 6 | 7 | 1 | 0 |
| *Layia carnosa* | Asteraceae | Annual Herb | Coastal Strand | Endangered | Endangered | 2 | 1 | 1 | 0 | 1 |
| *Layia heterotricha* | Asteraceae | Annual Herb | Grassland | None | None | 45 | 0 | 0 | 0 | 0 |
| *Layia jonesii* | Asteraceae | Annual Herb | Chaparral | None | None | 12 | 1 | 2 | 1 | 1 |
| *Lonicera subspicata var. subspicata* | Caprifoliaceae | Perennial Shrub | Chaparral | None | None | 13 | 0 | 1 | 1 | 0 |
| *Lupinus nipomensis* | Fabaceae | Annual Herb | Coastal Strand | Endangered | Endangered | 7 | 0 | 0 | 0 | 0 |
| *Malacothamnus palmeri var. involucratus* | Malvaceae | Perennial Shrub | Chaparral | None | None | 4 | 0 | 0 | 0 | 0 |
| *Malacothamnus palmeri var. palmeri* | Malvaceae | Perennial Shrub | Chaparral | None | None | 8 | 1 | 2 | 0 | 1 |
| *Malacothrix similis* | Asteraceae | Annual Herb | Coastal Sage Scrub | None | None | 1 | 0 | 1 | 0 | 0 |
| *Microseris paludosa* | Asteraceae | Perennial Herb | Northern Coastal Scrub | None | None | 1 | 0 | 1 | 0 | 0 |
| *Mimulus fremontii var. vandenbergensis* | Phrymaceae | Annual Herb | Chaparral | Candidate | None | 11 | 0 | 0 | 0 | 0 |
| *Monardella crispa* | Lamiaceae | Perennial Herb | Coastal Strand | None | None | 28 | 3 | 5 | 4 | 5 |
| *Monardella frutescens* | Lamiaceae | Perennial Herb | Coastal Strand | None | None | 27 | 6 | 7 | 4 | 2 |
| *Monardella palmeri* | Lamiaceae | Perennial Herb | Chaparral | None | None | 12 | 1 | 1 | 0 | 0 |
| *Monolopia gracilens* | Asteraceae | Annual Herb | Forest | None | None | 5 | 2 | 2 | 0 | 0 |
| *Nasturtium gambelii* | Boraginaceae | Perennial Herb | Freshwater Marsh | Endangered | Threatened | 9 | 1 | 2 | 1 | 0 |
| *Orobanche parishii ssp. brachyloba* | Orobanchaceae | Perennial Herb | Coastal Strand | None | None | 1 | 1 | 1 | 1 | 0 |
| *Pentachaeta lyonii* | Asteraceae | Annual Herb | Chaparral | Endangered | Endangered | 21 | 0 | 0 | 0 | 0 |
| *Pinus radiata* | Pinaceae | Perennial Tree | Forest | None | None | 1 | 0 | 1 | 0 | 0 |
| *Sanicula maritima* | Apiaceae | Perennial Herb | Prairie | None | Rare | 12 | 0 | 0 | 0 | 0 |
| *Scrophularia atrata* | Scrophulariaceae | Perennial Herb | Coastal Strand | None | None | 44 | 0 | 3 | 1 | 2 |
| *Senecio aphanactis* | Asteraceae | Annual Herb | Forest | None | None | 10 | 1 | 1 | 1 | 1 |
| *Streptanthus albidus ssp. peramoenus* | Boraginaceae | Annual Herb | Chaparral | None | None | 18 | 2 | 2 | 1 | 0 |
| *Suaeda californica* | Chenopodiaceae | Perennial Shrub | Salt Marsh | Endangered | None | 9 | 5 | 6 | 3 | 1 |
| *Suaeda esteroa* | Chenopodiaceae | Perennial Herb | Salt Marsh | None | None | 3 | 2 | 3 | 1 | 0 |
| *Sulcaria isidiifera* | Parmeliaceae | ??? ??? | ??? | None | None | 2 | 1 | 1 | 0 | 0 |
| *Symphyotrichum defoliatum* | Asteraceae | Perennial Herb | Forest | None | None | 1 | 0 | 0 | 0 | 0 |
| *Thelypteris puberula var. sonorensis* | Thelypteridaceae | Perennial Fern | Marsh | None | None | 10 | 0 | 1 | 0 | 1 |
| *Trifolium hydrophilum* | Fabaceae | Annual Herb | Marsh | None | None | 1 | 0 | 0 | 0 | 0 |
